# Membranous nephropathy: pathogenesis and treatments

**DOI:** 10.1002/mco2.614

**Published:** 2024-06-29

**Authors:** Mengqiong Wang, Jingjuan Yang, Xin Fang, Weiqiang Lin, Yi Yang

**Affiliations:** ^1^ Department of Nephrology Center for Regeneration and Aging Medicine The Fourth Affiliated Hospital of School of Medicine and International School of Medicine, International Institutes of Medicine Zhejiang University Yiwu China

**Keywords:** antigenic epitope, HLA, kidney pathogenesis, membranous nephropathy, PLA2R

## Abstract

Membranous nephropathy (MN), an autoimmune disease, can manifest at any age and is among the most common causes of nephrotic syndrome in adults. In 80% of cases, the specific etiology of MN remains unknown, while the remaining cases are linked to drug use or underlying conditions like systemic lupus erythematosus, hepatitis B virus, or malignancy. Although about one‐third of patients may achieve spontaneous complete or partial remission with conservative management, another third face an elevated risk of disease progression, potentially leading to end‐stage renal disease within 10 years. The identification of phospholipase A2 receptor as the primary target antigen in MN has brought about a significant shift in disease management and monitoring. This review explores recent advancements in the pathophysiology of MN, encompassing pathogenesis, clinical presentations, diagnostic criteria, treatment options, and prognosis, with a focus on emerging developments in pathogenesis and therapeutic strategies aimed at halting disease progression. By synthesizing the latest research findings and clinical insights, this review seeks to contribute to the ongoing efforts to enhance our understanding and management of this challenging autoimmune disorder.

## Graphical abstract

Membranous nephropathy, an autoimmune disease, can manifest at any age and is among the most common causes of nephrotic syndrome in adults. This review explores recent advancements in the pathophysiology of MN, encompassing pathogenesis, clinical presentations, diagnostic criteria, treatment options, and prognosis, with a focus on emerging developments in pathogenesis and therapeutic strategies aimed at halting disease progression

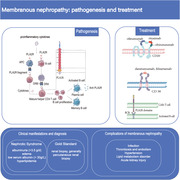

## 1 INTRODUCTION

**FIGURE 1:**
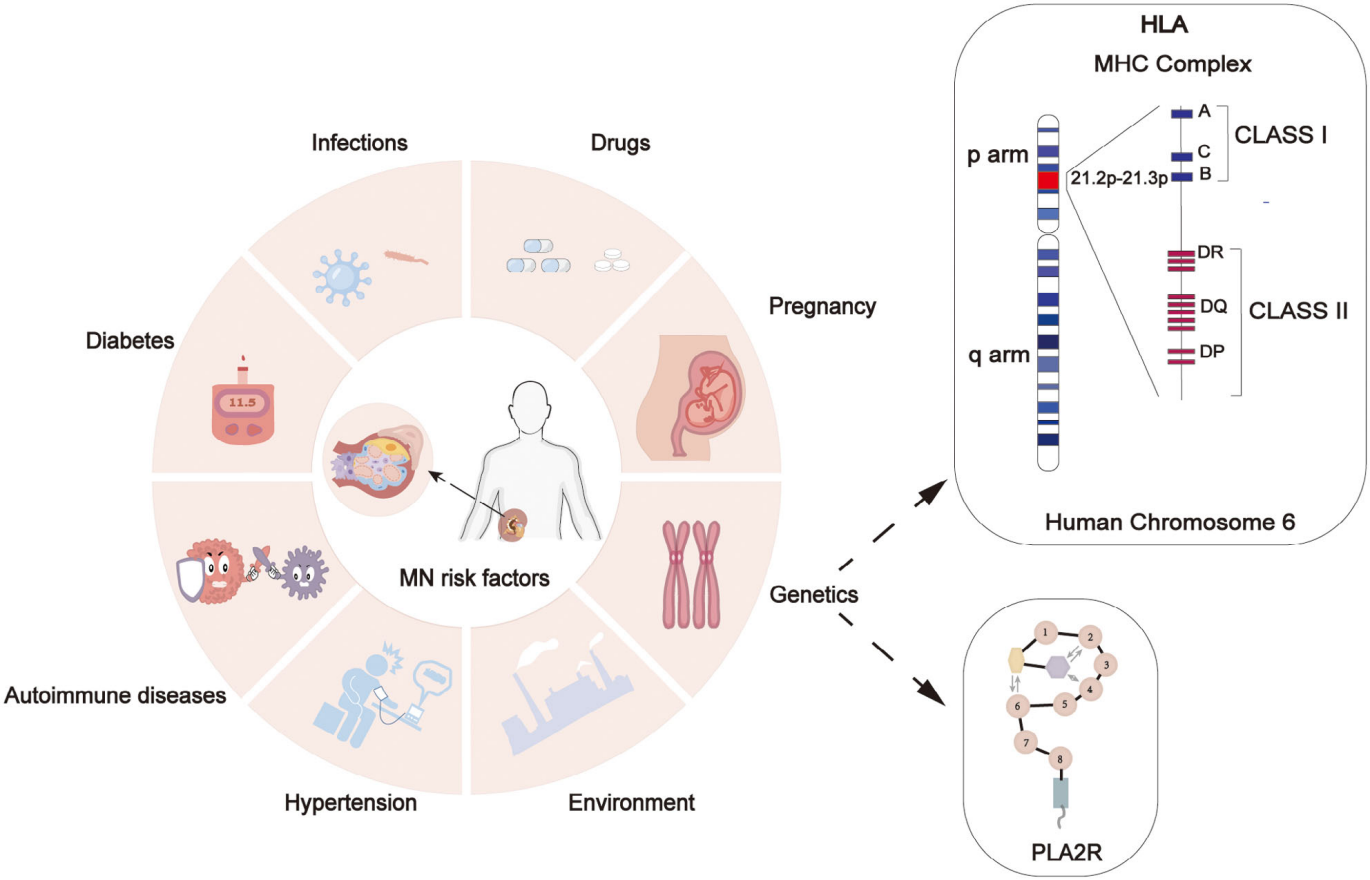
Membranous nephropathy risk factor. Membranous nephropathy can be caused by drugs, infections, diabetes, autoimmune diseases, hypertension, pregnancy, environment, and genetics. The interaction between HLA and PLA2R may contribute to the development and progression of membranous nephropathy.

Membranous nephropathy (MN), also known as membranous glomerular disease,^1^ is a distinct autoimmune glomerular disorder^2^, ^3^ and stands as the most prevalent cause of nephrotic syndrome in adults.^4^, ^5^, ^6^ It was proposed by Wells and Bell^7^ and identified as a distinct pathological entity by Jones^8^ in 1957. The global incidence of the disease is estimated to be 8−10 cases per 1 million individuals,^6^, ^9^ with China reporting an incidence of 23.4%, ranking second only to IgA nephropathy and showing a year‐on‐year increase.^10^, ^11^, ^12^ MN can manifest at any age, with the average age at diagnosis falling between 50−60 years,^13^ and a notably higher incidence among males than females.^14^ About 75% of patients are classified as primary membranous nephropathy (PMN), with no discernible underlying cause. The remaining 20% of cases may be linked to factors such as tumor development, infection, autoimmunity, or drug usage, categorized as secondary membranous nephropathy (SMN)^2^, ^15^ (Figure 1). The pathology of MN is characterized by global and diffuse distribution of subepithelial deposits,^16^, ^17^ leading to thickening of the capillary wall when examined under a light microscope.

Significant strides have been accomplished in elucidating the pathogenesis of MN in recent decades. The identification of key autoantigens such as phospholipase A2 receptor (PLA2R)^18^ and thrombospondin type‐1 domain‐containing 7A (THSD7A)^19^, ^20^ has substantially propelled both fundamental and clinical MN investigations, leading to a transformative shift from histological to pathophysiological perspectives in understanding this disease.^21^ With the discovery of additional autoantibodies and target antigens, there is a growing consensus among experts for a reassessment of MN classification.^22^ A comprehensive grasp of MN pathogenesis is essential for the development of effective treatment strategies. An in‐depth understanding of disease mechanisms enables clinicians to target specific pathological and physiological processes, thereby optimizing therapeutic outcomes. Notably, research has highlighted the pivotal role of B lymphocytes in MN pathogenesis,^23^ with surface molecules CD20^24^ and CD38^25^ identified as potential therapeutic targets, paving the way for the exploration of new immunosuppressive agents.

Currently, the therapeutic options for MN predominantly encompass a range of approaches, including supportive therapy, immunosuppressive therapy, and investigational targeted therapies. It is recommended that all patients with MN undergo maximally tolerated conservative therapy^22^ to alleviate clinical symptoms such as proteinuria, edema, and hypertension, with approximately 30% of patients experiencing spontaneous remission after supportive therapy.^26^, ^27^ For patients who do not achieve remission or are at higher risk of disease progression, immunosuppressive or combination therapies are often recommended.^28^ However, while these therapies can attenuate the immune system's assault on the glomeruli and slow disease progression, they may also elicit serious adverse effects on the body. In the future, targeted therapies tailored to individualized pathogenesis have the potential to supplant traditional immunosuppressive therapies, offering a more precise approach to treating MN.

In this review, we aim to present a comprehensive overview of the current understanding of the pathogenesis and treatment of MN, incorporating recent research advancements in crucial factors including PLA2R and HLA. We delve into the clinical manifestations, diagnostic approaches, and potential complications and prognosis associated with MN. Moreover, we offer practical recommendations aimed at enhancing renal prognosis and improving quality of life, thereby furnishing valuable guidance for clinical practitioners. This extensive review not only facilitates a profound comprehension of the pathophysiological mechanisms underlying MN but also serves as a significant reference for future research endeavors and clinical interventions.

## 2 PATHOGENESIS OF MN

In previous investigations, we have made significant strides in comprehending the pathophysiological mechanisms underlying MN by extensively exploring the rat Heyman MN model. These investigations have yielded invaluable insights, elucidating the pivotal role of podocytes and antigens in the pathogenesis of MN. In this section, we will concentrate on elucidating the involvement of autoimmunity and podocyte injury in the pathogenesis of MN, while also examining the impact of genetic and environmental factors on the disease. These components will enhance our understanding of the pathogenesis of MN but will also provide crucial guidance for future research endeavors and clinical interventions.

### 2.1 Autoimmune mechanisms underlying MN development

**FIGURE 2:**
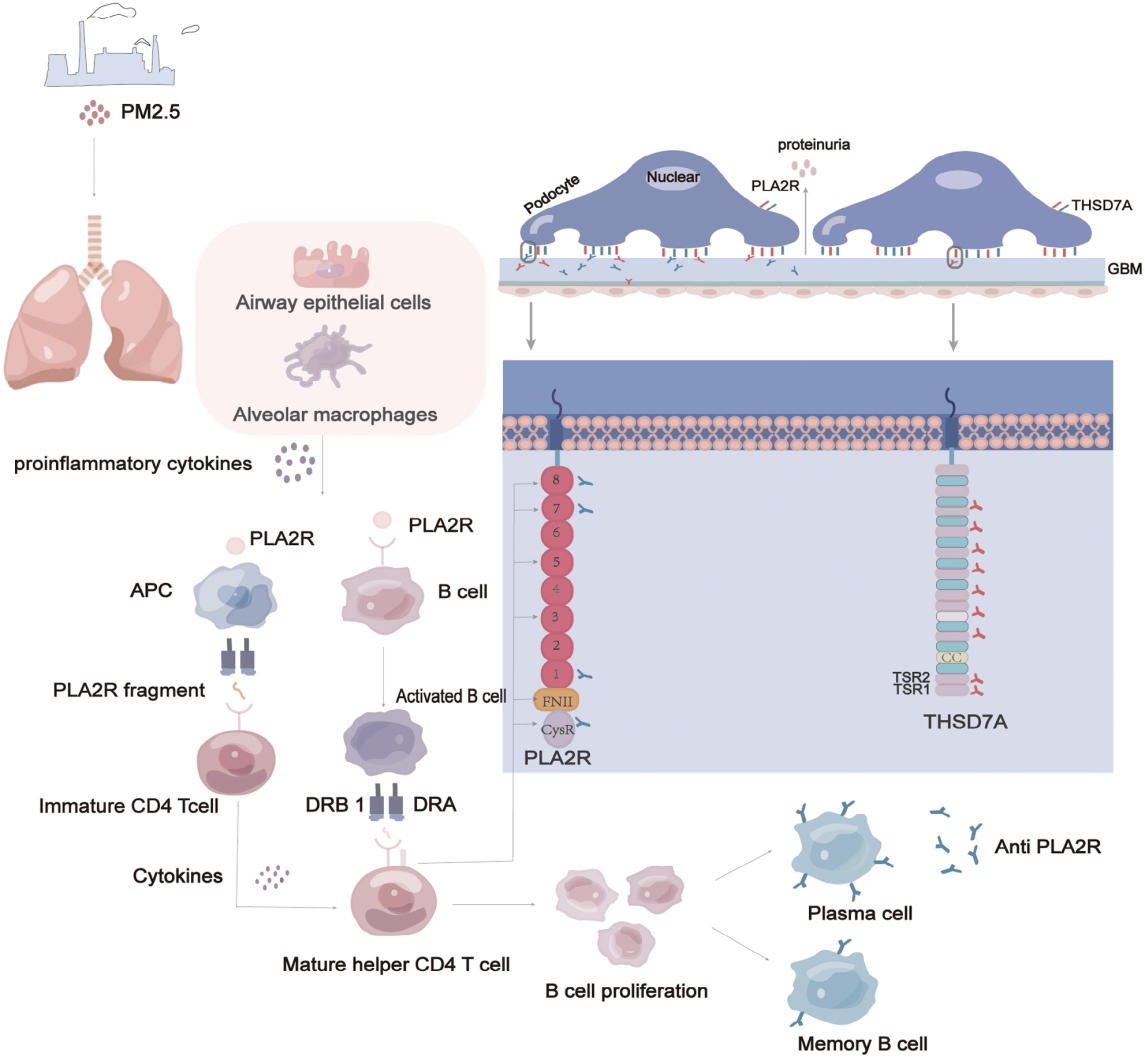
Mechanism for the development of membranous nephropathy. The pathogenicity of PLA2R in MN is associated with anti‐PLA2R antibodies produced in dependence on MHCII. Class II molecules expressed on antigen‐presenting cells present extracellular PLA2R fragments to CD4+ T cells through the antigen‐presenting groove formed by the membrane's α and β anchor chains. This leads to B cell activation, massive proliferation, and production of anti‐PLA2R antibodies. These antibodies can infiltrate the endothelium and GBM toward the PLA2R, forming antigen–antibody immune complexes.

MN represents an antibody‐mediated autoimmune condition wherein the body mounts an antibody response against glomerular antigens due to compromised immune tolerance, culminating in renal impairment.^29^ The pivotal demonstration of neutral endopeptidases (NEPs) in immune complexes in infants by Debiec et al.^30^ in 2002 provided critical substantiation for MN's autoimmune nature. Presently, two distinct autoimmune pathways underlie MN. One pathway encompasses antigen binding to autoreactive B cells, internalization, fragmentation, subsequent presentation to helper T cells via major histocompatibility complex (MHC) class II receptors, and release of cytokines from activated T cells, triggering feedback to B cells, fostering division and differentiation into plasma cells, and instigating antibody production and memory B cell formation. Another pathway entails antigen‐presenting cells, such as dendritic cells or macrophages, recognizing antigens, processing and presenting antigen fragments via MHC class II molecules, thereby activating self‐reactive T cells. Antigen‐T cell binding activates B cells, which then migrate to the germinal center, interact with helper T cells, and undergo proliferation and differentiation, leading to the generation of memory B cells or plasma cells^31^ (Figure 2). In summation, the pathogenesis of idiopathic membranous nephropathy (IMN) remains a complex multifaceted process necessitating a comprehensive comprehension.

**FIGURE 3:**
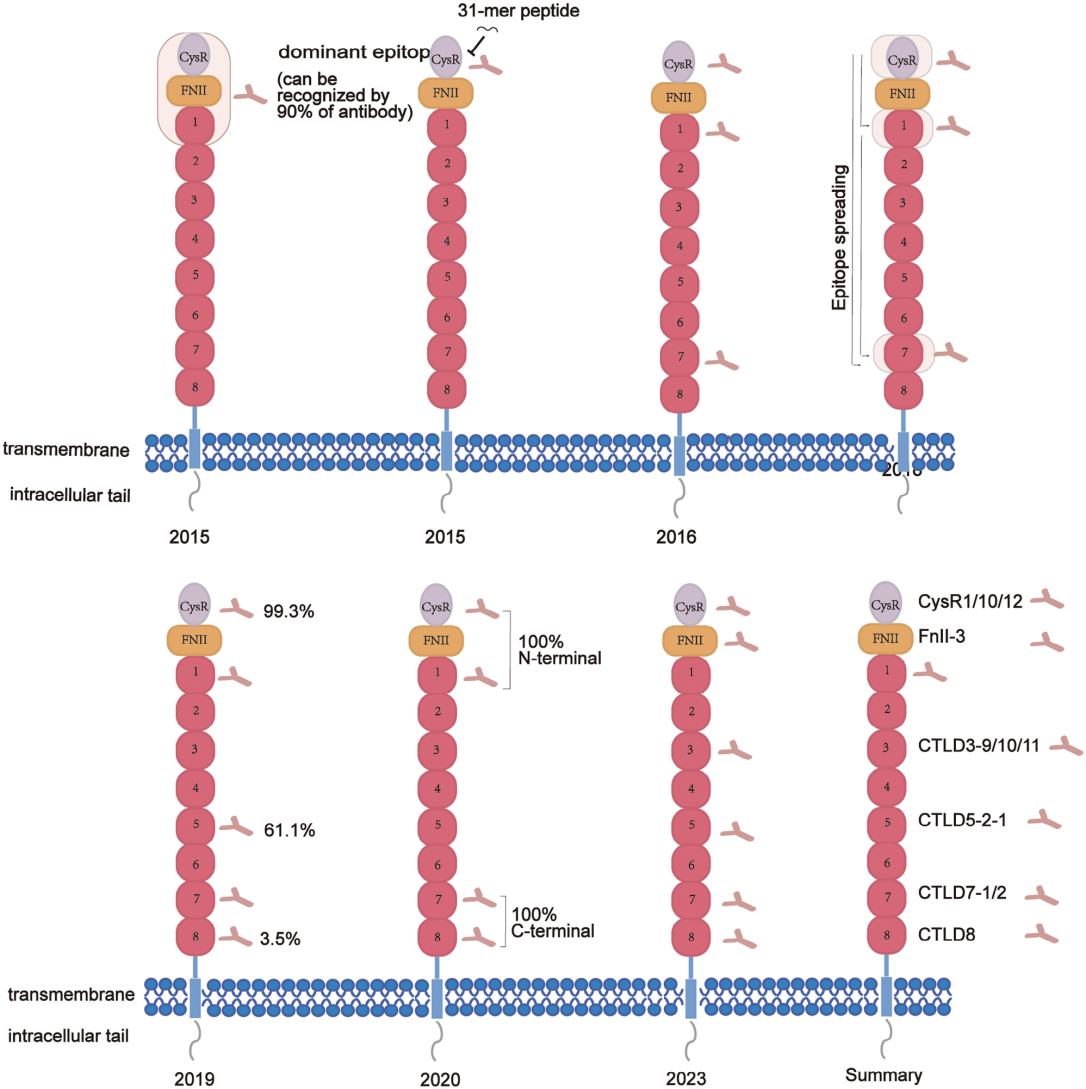
History of antigenic epitope discovery.

PLA2R, a type I transmembrane glycoprotein with a relative molecular weight of 180 kDa,^32^ is expressed in various organs, encompassing the lung, placenta, and liver. However, it is primarily found in the glomerular podocytes, with about 70−80% of MN patients showing the presence of anti‐PLA2R antibodies.^33^ It is notable in upholding the phospholipid composition of cell membranes and regulating apoptosis in foot cells.^34^ Beck et al. made the seminal discovery of PLA2R in 2009.^18^ They isolated glomeruli from the kidneys of IMN patients using a hierarchical screening method and performed protein‐botting immunoblot analysis of extracts from serum samples under nonreducing conditions. About 70% of the patient sera recognized a 185 kDa protein, which was subsequently examined by employing a liquid chromatography‐tandem mass spectrometry technique, identifying the protein as M‐type PLA2R.^18^ This landmark discovery transformed our understanding of membrane nephropathy, confirmed the autoimmune nature of idiopathic membrane nephropathy, and established a foundation for exploring membrane nephropathy's pathogenesis. This receptor comprises a single transmembrane helix encompassing 10 extracellular structural domains, specifically the cysteine‐rich domain (CysR), the fibronectin type II domain (FnII), and eight C‐type lectin domains (CTLDs).^35^, ^36^ In 2016, Seitz‐Polski et al.^37^ discovered all CysR epitopes to be positive in serum samples from 50 anti‐PLA2R positive patients, confirming CysR as the predominant dominant epitope. Detailed mapping of other epitopes revealed that in addition to CysR and CTLD1, a third independent reactive epitope exists in CTLD7. Peptides 285 (SKTVEVWMGLNQLDE) from the structural domain of CTLD1, peptides 1130 (NANMTWYAAIKTCLM) and 1194 (SFTFWKDEESSLLGD) from the structural domain of CTLD7, along with peptide 815 (PWLFYQDA) from the region between CTLD4 and CTLD5, were potential targets for autoantibodies. In 2019, Justino^38^ further investigated the number of epitopes in the distal region of PLA2R1. They constructed a series of individual structural domains. They tested their reactivity via ELISA experiments on 144 MN patients, identifying CTLD5 and CTLD8 as two new epitope‐containing structural domains besides the CTLD7 structural domain. Of these, the CysR structural domain was the most prevalent epitope‐containing structural domain (99.3%), while CTLD5 was the second most prevalent (61.1%), and CTLD8 was the least prevalent (3.5%). Reinhard's team identified a fourth independent antigenic epitope in CTLD8 in 2020. Further, it was shown that all patients had antibodies against the N‐terminal (CysR or CTLD1) epitope. Although CTLD2 and CTLD3 do not directly bind ligands, their interactions with one another in a pH‐dependent manner are critical for conformational changes in the N‐terminal region of CysR that may influence ligand binding or release during autoimmunity.^39^ A recent research by Tang and colleagues^40^ demonstrated that anti‐PLA2R antibodies could bind to denatured CysR‐CTLD3 and CysR‐CTLD1 structural domains differently. Notably, as of 2022, the results of foreign antigenic epitope studies have yet to be validated in the Chinese population. Further, while all previously reported epitopes were B‐cell epitopes, an understanding of T‐cell epitopes of PLA2R remains to be studied. A recent study conducted by Cui and coworkers^41^ has identified a total of 17 PLA2R peptides that demonstrate significant binding affinity towards DRB1*1501 and DRB1*0301 molecules. Reduced binding was seen for several of these peptides to the heterodimeric DRB1*1501/0901 and DRB1*0301/0701. Among these peptides, 10 sequences, namely, PLA2R38‐52 (CysR1), PLA2R101120 (CysR10), PLA2R113‐129 (CysR12), PLA2R193‐212 (FnII‐3), PLA2R602‐621 (CTLD3‐9), PLA2R612‐631 (CTLD3‐10), PLA2R622‐641(CTLD3‐11), PLA2R829‐838(CTLD5‐2‐1), PLA2R1121‐1140 (CTLD7‐1), and PLA2R1129‐1150 (CTLD7‐2), have been identified as potential T cell epitope‐binding HLA‐DRB risk molecules, upon activation of these peptides, peripheral blood mononuclear cells obtained from MN patients exhibited an increase in proinflammatory cytokine levels, specifically IL‐6, TNF‐α, IL‐10, IL‐9, and IL‐17, which in turn enhanced immunoglobulin synthesis by stimulating T cell proliferation, promoting B cell activation and proliferation, and differentiating cells towards plasma cells^42^ (Figure 3). However, the limitation of this study is that only peptides with a high binding capacity to HLA risk genes were selected, which may ignore the potentiality of peptides with moderate binding capacity as T cell epitopes. Additionally, the precise mechanism governing the interaction of PLA2R antibodies with the conformational epitopes of PLA2R remains ambiguous and necessitates further elucidation.

In addition to PLA2R, other antigens, such as NEP and THSD7A, have been found in MN. Alloimmune antibodies against NEP^43^ were found in pregnant women with NEP deficiency, resulting in the development of immune complexes in the fetal glomerular basement membrane (GBM). THSD7A is a 250 kDa protein found in blood samples from individuals with IMN who test negative for anti‐PLA2R1 antibodies.^44^ Cross‐reactivity between PLA2R and THSD7A autoantibodies has been observed at the peptide level,^45^ although THSD7A‐associated cases are rare, accounting for only 2−5% of IMN.^46^ In recent years, other autoantibodies targeting antigens, such as neural tissue encoding a protein with epidermal growth factor (EGF)‐like repeats (NELL1),^47^, ^48^ exostosin 1/exostosin 2 (EXT1/EXT2),^49^ semaphorin 3B (SEMA3B),^50^ protocadherin 7 (PCDH7),^51^ neural cell adhesion molecule (NCAM1),^52^ protocadherin FAT1 (FAT1),^53^ neuron‐derived neurotrophic factor (NDNF),^54^ reticulin G1,^55^ FCN,^56^ transforming growth factor beta receptor 3 (TGFBR3),^57^ high‐temperature recombinant protein A1 (HTRA1),^58^ and contact protein‐1 (CNTN1)^59^ have also been identified by researchers from China and other countries. This extensive library of target antigens has led to the proposal of a new antigen‐based classification system that can increase the accuracy and specificity of the diagnosis of MN.^60^, ^61^ The differential expression levels of these antigens in MN and LN glomeruli provide new insights into their pathogenesis.^62^ Proprotein convertase subtilisin/kexin type 6 (PCSK6) is a new protein that Sethi et al.^63^ discovered using laser microdissection and tandem mass spectrometry. They demonstrate that PCSK6 is the likely target antigen in MN in patients who have used NSAIDs for a lengthy period. Precise characterization of PLA2R antigenic epitopes in MN patients assumes paramount significance in the development of novel antigen‐ or epitope‐specific therapies; hence, future research should strive to uncover additional new antigens and antigenic epitopes. While therapeutic strategies targeting PLA2R antigenic epitopes are still in the early stages of investigation, further validation of their therapeutic efficacy is warranted.

### 2.2 Role of podocyte injury in the pathogenesis of MN

Podocytes, highly specialized epithelial cells, exhibit a distinctive tripartite structure comprising the cell body, major protrusion, and peduncle. The cell body is rich in organelles including the nucleus, endoplasmic reticulum, Golgi apparatus, lysosomes, and mitochondria. Predominant within the glomerulus, it plays a central role in renal function. Attached to the outer layer of the GBM,^64^ the peduncle is pivotal in the renal filtration barrier, engaging with intercellular junctions and matrix proteins.^65^, ^66^ Notably, the apical domains of the peduncle bear a negative charge, facilitating the retention of negatively charged molecules like albumin and preserving the integrity of neighboring peduncles through anionic charge separation.^67^ As a special epithelial cell of the glomerulus, podocytes surround the glomerular capillary wall and have special shapes, structures and functions. The dynamic actin filament network in cells can regulate foot process tension, help relieve the impact of high pressure in capillaries on the filtration membrane, and maintain the structural stability.^68^ The slit diaphragm formed between the secondary processes of adjacent podocytes is a key part in determining whether substances in the blood are filtered into the urine. Under normal physiological conditions, only water, electrolytes, and small molecule substances are allowed to filter, unlike other structures. Together they maintain selective filtering function.^69^ Cytoskeletal components of podocytes, such as actin filaments, can control the size and permeability of filtration clefts to adapt to changes in renal hemodynamics under different physiological states.^70^ In addition, podocytes are also involved in the synthesis and secretion of GBM components, including the expression of a variety of proteins (such as nephrin, podocin, etc.).^71^ Focal adhesions expressed by podocytes, in addition to adhesion, also relay intracellular signaling pathways, maintaining the balance between the intracellular and extracellular signals, and affecting the biological behavior of glomerular endothelial cells and mesangial cells, and overall renal homeostasis.^72^

Additionally, the foot cell, beyond its role in stabilizing cytoskeletal morphology and sustaining biological function, emerges as a significant and distinctive target of the autoimmune response.^73^ It possesses the capacity to present endogenous antigens like PLA2R and THSD7A or create an environment favorable for the deposition of exogenous antigens such as those from hepatitis B and C viruses.^74^ The low molecular mass of HBeAg, with its negative charge, can be embedded in the epithelial side of the GBM, initiating the formation of immune complexes in situ. Podocytes demonstrate the ability to express MHC class I and II antigens,^75^, ^76^ uptake soluble and granular antigens, activate CD4+ T cells, and cross‐present exogenous antigens on MHC class I molecules to CD8+ T cells. Studies have revealed the expression of B7‐1 (CD80),^77^ an immunoglobulin superfamily member, on podocytes, with its aberrant expression induced in the presence of hypoxia, high glucose, or bacteriocin lipopolysaccharide, leading to cytoskeletal reorganization, morphological alterations in podocytes, and increased GBM permeability. The adhesion between podocytes and GBM is regulated by various integrins, which are cytoskeletal adhesion receptors located on the cell membrane. Talin‐1, a cytoskeletal protein, mediates integrin‐actin binding and interacts with β3 integrins, yet an inhibitor of B7‐1 disrupts this interaction, resulting in podocyte detachment.^78^

**FIGURE 4:**
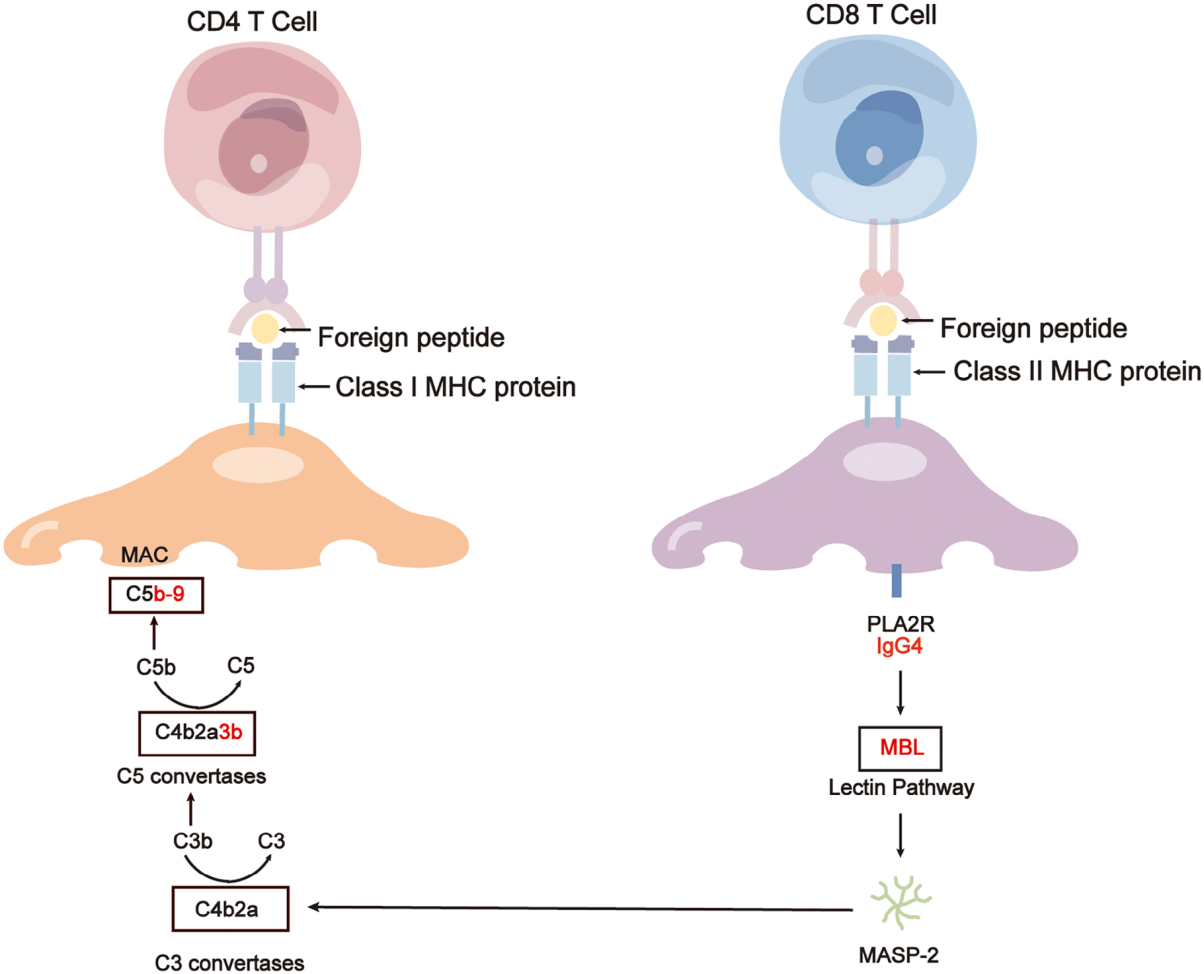
Role of podocyte injury in the pathogenesis of MN. Podocytes have the capability to express major histocompatibility complex (MHC) class I and class II antigens uptake both soluble and particulate antigens, activate CD4+ T cells, and engage in cross‐presentation of exogenous antigens on MHC class I molecules to CD8+ T cells. In PMN, anti‐PLA2R1 IgG4 can activate complement in a glycosylation‐dependent manner. This complement activation leads to the formation of membrane attack complexes (MAC, C5b‐9) on podocytes, resulting in podocyte sublytic injury or inducing podocyte pyroptosis, ultimately disrupting the glomerular filtration barrier and causing proteinuria. MBL, Mannose‐binding lectin.

Cells maintain homeostasis in vivo, ensuring a healthy state. Podocytes, when exposed to physiological stresses such as the shear stress of ultrafiltrate, demonstrate adaptability through their unique mechanical biology to sustain function.^79^ However, when these mechanical stresses surpass the podocyte's tolerance threshold, this biological equilibrium is disrupted, resulting in the loss of glomerular function. Podocyte injury stems from various factors, including mechanical, oxidative, and immune stresses, with the formation of subepithelial immune complexes and complement activation being pivotal.^80^ These immune deposits comprise components such as IgG (typically IgG4),^81^, ^82^, ^83^ antigens, and membrane attack complexes (MACs). In PMN, anti‐PLA2R1 IgG4 triggers complement activation in a glycosylation‐dependent manner,^84^ leading to the formation of MACs (C5b‐9) on podocytes.^85^ C5b‐9 activation initiates diverse downstream pathways within the podocyte, encompassing protein kinases, lipid metabolism, cytokine production, ROS generation, growth factor signaling, endoplasmic reticulum stress, and the ubiquitin–proteasome system. These actions result in sublytic damage to podocytes or induce podocyte ferroptosis, ultimately compromising the integrity of the glomerular filtration barrier and culminating in proteinuria.^86^ The slit diaphragm represents the ultimate and primary barrier against protein entry into the urinary filtrate, with the podocyte cytoskeleton orchestrating the slit molecules. Consequently, targeted therapeutic strategies directed towards podocyte agonist proteins hold promise as potential breakthrough treatments for renal diseases in the future^87^ (Figure 4).

Podocytes lack proliferative potential and are susceptible to damage and loss under various stimuli. The primary clinical manifestation of podocyte damage is proteinuria, often accompanied by renal function decline and glomerulosclerosis progression. Thus, the principal therapeutic aim should focus on repairing or replacing damaged or lost podocytes. Current immunotherapeutic agents, such as glucocorticoids and calcium‐modulated phosphatase inhibitors, exhibit direct effects on podocytes. However, the effective delivery and modulation of podocyte‐specific therapies present significant technical hurdles. A comprehensive understanding of the factors and mechanisms underlying podocyte injury is crucial for preventing MN and identifying novel therapeutic targets.

### 2.3 Genetic and environmental factors contributing to MN development

Despite MN not being a typically inherited disease, extensive research over the past decade has increasingly demonstrated its susceptibility to be closely linked to genetic factors.^88^ Over the past decade, genome‐wide association studies (GWAS) have emerged as a powerful technique for understanding the genetic basis of complex human traits, providing many mindful perspectives on understanding MN. A GWAS revealed that the HLA locus on chromosome 6 and the PLA2R locus on chromosome 2 are significantly associated with MN.^89^

HLA was initially discovered in human leukocytes in 1958.^90^ It constitutes a highly polymorphic complex comprising intricate interlocking motifs. A study unveiled a robust correlation between the HLA‐A1/B8/DR3 haplotype and IMN, laying the groundwork for subsequent investigations into the HLA locus and MN.^91^ While HLA‐DQ and HLA‐DR alleles have been identified as risk factors, their specific loci vary across ethnicities. Vaughan et al.^92^ in 1995 demonstrated a positive association between HLA‐DQA1*0501 and an elevated IMN risk in Greek and British populations. Further research revealed HLA‐DRB*0301 as weakly correlated with the Greek population.

In the Chinese population, DRB1*15:01, DRB1*0301,^93^ and DRB3*02:02^94^ were strongly associated with PLA2R‐related MN, while in the Japanese population, HLA‐DRB1*1501 and DQB1*0602 were notably linked to MN.^95^ Additionally, Le et al.^96^ identified 79 additional HLA isoforms in 392 PLA2R‐positive Chinese Han patients, elucidating four HLA alleles—DRB1*1301, DQB1*0603, DRB1*0405, and DQB1*0302—as independently associated with a poor prognosis in MN patients, leading to a ≥40% decrease in estimated glomerular filtration rate (eGFR) during follow‐up.

Single nucleotide polymorphisms (SNPs) represent genetic variations arising from unique nucleotide alterations in the genomic sequence, marking the third generation of genetic markers following restriction endonuclease fragment length polymorphisms, variable repeat sequences, and microsatellite polymorphisms. SNPs located within the HLA‐DQA1 locus exert significant influence on PMN across various racial populations. In 2011, Stanescu et al.^89^ conducted a genomic analysis involving 556 patients diagnosed with IMN, revealing a strong correlation between risk alleles within the HLA‐DQA1 locus and IMN prevalence in European populations. These alleles may incite an autoimmune response targeting specific entities such as PLA2R1 antibodies, potentially acting as protective factors in preserving renal function.^89^ Notably, the SNPrs2187668 locus of the HLA‐DQA1 gene has been identified as a susceptibility locus for IMN in Caucasian, Hispanic, and Indian populations in Europe, but not in South China.^97^ Furthermore, the HLA‐DQA1 gene has been recognized as a risk allele for IMN in the European population. Independent investigations in India^98^ and China^99^ have corroborated these findings, associating the HLA‐DQA1 SNP rs2187668 AA and AG variants with heightened susceptibility. This genetic variant presents opportunities for novel therapeutic interventions in IMN management. Regulating this gene mutation offers promising avenues for innovative IMN therapies. Notably, the UK population exhibits the highest MN prevalence, particularly among individuals harboring the high‐risk HLA‐DQA1 allele (TT).^100^ Additionally, two SNPs, rs9271705 and rs9271550, demonstrate significant associations with MN recurrence in transplant recipients.^101^

The recognition of PLA2R as a significant antigen in MN has spurred investigations into genetic susceptibility to the condition. In particular, GWAS have demonstrated that PLA2R SNPs, including rs3749117, rs3792189, rs3792192, rs6722275, rs1870102, rs271592, and rs35771982,^102^, ^103^ are significantly associated with European IMN. Subsequently, research on PLA2R gene polymorphisms has extended to multiple countries. For example, studies within the Japanese population identified significant correlations for rs35771982, rs3749119, rs2715928, rs16844715,^95^ rs3749117, rs2715918,^104^ as well as rs35771982, rs3749117, rs4664308 in the Chinese population,^97^, ^105^ while rs3749119,^106^ rs35771982, and rs38283223 demonstrated significance in the Korean population.^107^ Among South Asian and East Asian populations, rs3749119, rs3749117 and rs4664308,^98^ rs230540 (NFKB1), and rs9405192 (IRF4)^108^ were correlated with IMN. Furthermore, rs4665143, rs749117, rs35771982, and rs3828323^109^ demonstrated significant associations in the French population. Significantly, it has been postulated that pathogenic or inducible mutations in the PLA2R sequence could potentially induce alterations in the protein's structure.^31^ Additionally, amino acid substitutions within distinct variants may result in the creation of a peptide sequence that exhibits a greater affinity for HLA‐DQA1 binding, consequently facilitating the display of the mutated domain on the cell surface. By doing so, T cells acquire the ability to monitor this region. Nevertheless, it is important to acknowledge that sequence variations, particularly changes in the CTLD region, might also impact the functionality of PLA2R1.^31^ These functional alterations can include influences on extracellular matrix organization, endocytosis, complement activation, and intercellular interactions.^110^, ^111^ However, no studies have provided sufficient evidence to support this hypothesis. Coenen et al.^109^ explored PLA2R gene polymorphisms more profoundly and found nine common variants on the PLA2R gene, among which three SNP loci encoding nonsynonymous substitutions, rs3749117, rs35771982, and rs3828323, could affect the multidimensional stereospecific structure of PLA2R, which in turn altered the sensitivity, specificity, and affinity of PLA2R to autoantibody binding sensitivity, specificity, and affinity, and concluded that the G allele of rs 35771982 is the susceptible allele, a result consistent with the findings of Liu et al.^99^ in Taiwan residents and Lv et al.^105^ and Indian^98^ populations found the AA genotype at the rs4664308 locus of the PLA2R gene to be the risk genotype, Kim et al.^107^ concluded that the CC genotype at the rs35171982 locus was the risk genotype for IMN in Koreans and was not associated with SMN, and the risk allele genotype in Japanese populations was C at rs35771982, G at rs2715928, and T at rs16844715. PLA2R and HLA gene polymorphisms could explain why certain individuals with PMN experience unanticipated resolution, whereas others display a recurring relapse pattern.^112^

HLA class II genes, which encode HLA‐DR, ‐DP, and ‐DQ molecules, present epitopes to CD4‐positive T cells, subsequently differentiating into helper T cells and activating diverse immune responses. In 2016, Cui et al.^93^ elucidated a structural model wherein amino acids encoded by DRB*1501 and DRB*0301 facilitate PLA2R's T cell epitopes. With the identification of an interaction between PLA2R1 and specific HLA alleles, researchers have further discerned that this interaction augments genetic susceptibility to MN. Moreover, the escalating economic development has led to increased public health concerns related to heavy metals, organic compounds, and air pollution, correlating with a rise in various diseases.^113^ Studies indicate that prolonged exposure to elevated concentrations of PM2.5 heightens the risk of MN development,^114^ potentially attributable to the augmentation of autoantibodies and immune complexes by fine particulate matter.^115^ The lungs are large and susceptible to environmental factors. PM2.5 inhalation can cause inflammation in the lungs, leading to the accumulation of inflammatory vesicles in the airways or alveoli, which allows the release of PLA2R, which is expressed on neutrophils and macrophages, into the circulation, initiating an autoimmune response^116^ (Figure 2). Nonetheless, this study possesses certain limitations, necessitating further exploration into the precise molecular mechanisms involved.

It is crucial to continue exploring the interplay between PLA2R, HLA, and novel pathogenic variants in MN. In‐depth elucidation of these interactions' underlying mechanisms and functional consequences will enhance our understanding of the disease's heterogeneity and potentially facilitate the development of more effective therapeutic approaches. Furthermore, integrating genomic data with clinical information and longitudinal follow‐up will enable the identification of prognostic markers and personalized treatment strategies. Ultimately, these emerging insights can improve patient outcomes and alleviate the burden of MN on affected individuals.

## 3 CLINICAL MANIFESTATIONS AND DIAGNOSIS

### 3.1 Signs and clinical symptoms

MN, is a chronic progressive kidney disease that usually has an insidious onset and is asymptomatic in the early stages or with only slight limb edema.^117^, ^118^ The clinical symptoms of most adult patients, whether primary or secondary MN, present mostly nephrotic syndrome, including albuminuria (>3.5 g/d), edema, low serum albumin (<30 g/L), and hyperlipidemia,^119^ with or without microscopic hematuria, while a few others only show protein loss on urinalysis (≤3.5 g/d).^120^, ^121^, ^122^ Compared with adults, pediatric patients tend to have secondary, atypical MN and a better prognosis, but they are more likely to have microscopic or gross hematuria in addition to nephrotic syndrome.^123^ Some patients will have concurrent hypertension, which correlates with the severity of MN, but it is rare to develop severe hypertension or for it to be present from the time MN is diagnosed. Patients may also experience nonspecific symptoms such as anorexia, nausea, malaise, fatigue, and infections.^118^ Although MN patients’ clinical features are typical as mentioned, diagnosis based on those phenotypes are often inadequate and requires more precise approaches.

### 3.2 Kidney biopsy and histopathology

As a familiar diagnostic and investigative modality for nephrologists, and the “gold standard” for many kidney diseases, renal biopsy, generally percutaneous renal biopsy, is the most direct and standardized method for diagnosing MN.^124^ The histopathologic information provided by renal biopsy can help to confirm and differentiate the type of MN, and suggest its staging.

The light microscopic appearance of PMN may be normal in the early stage, but as the disease progresses, the change of wrinkled, irregularly shaped spike‐like GBM and its later diffuse thickening (PASM staining) may be observed, which may also be accompanied by focal renal tubular atrophy, and occasional formation of crescents.^125^, ^126^ Immunofluorescence microscopy shows diffuse deposition of immunoglobulin IgG (usually IgG4) along the glomerular capillary wall, and most patients are also accompanied by C3 deposition.^127^, ^128^, ^129^ Under electron microscopy, in addition to irregular thickening of GBM, electron‐dense deposition can be seen between the outer GBM and epithelial cells with foot process effacement (podocyte fusion or retraction).^130^ PLA2R staining of renal biopsy samples from patients with seronegative PLA2R antibody is helpful for the diagnosis of PLA2R‐related MN.^56^

Light microscopy of MN induced by autoimmune disease or malignancy demonstrates glomerular capillary endothelial or mesangial proliferation, which is rarely seen in pMN.^131^ Precipitated IgG stained by immunofluorescence in SMN is usually of the three subtypes IgG1, IgG2, and IgG3 accompanied by C1q, IgA, and IgM.^132^ Under the submicroscopic structure, the GBM thickens after stimulation, wrapping the dense electron deposits under the epithelium and endothelium. After the deposits are removed, the GBM forms vacuole‐like holes, and these cavities are subsequently filled with GBM‐like substances.^130^, ^133^

Although the pathological microscopic findings are as described above in most cases, the situation is not absolute. For example, there are cases showing SMN in children caused by mercury poisoning. Despite the deterioration of renal function to stage IV chronic kidney disease, under the light microscope no thickening or spiking of the basement membrane.^134^

The Fogo team divided MN into four stages based on the histopathological findings of the biopsy. In stage I, there are no obvious visible changes under the light microscope, no obvious thickening of the GBM, and there may be a small amount of small electron dense objects under the epithelial cells. In stage II, there are more electron‐dense deposits under the epithelial cells, and the GBM is irregularly thickened to form “spike” between the dense objects. Stage III: GBM diffusely thickens and surrounds electron‐dense materials, and part of the electron‐dense materials is absorbed. Stage IV: The GBM thickens significantly, and the sediments in the GBM are absorbed and reduced, causing it to appear “worm‐eaten”.^135^ However, pathological manifestations are not parallel to disease severity and prognosis, and other clinical manifestations need to be taken into consideration.^136^

### 3.3 Clinical guidelines of kidney biopsy

Renal biopsy can provide a more accurate picture of the pathological conditions of the tissue taken, help to identify the type of MN and determine the progression of the disease, and facilitate the determination of the subsequent treatment plans and prognosis estimation. However, as an invasive procedure, renal biopsy carries certain risks, such as bleeding, perirenal hematoma, microscopic or gross hematuria, infection, and so on.^137^, ^138^, ^139^ Therefore, how to determine whether a patient should undergo this operation and the timing of the operation deserve attention.

In general, renal biopsy is indicated in patients with PMN presenting with proteinuria >3 g/d or proteinuria <3 g/d but with rapidly increasing Scr levels and new‐onset haematuria.^124^ In patients with SMN, if renal function is impaired or combined with other renal disorders (such as diabetic nephropathy, crescentic glomerulonephritis), taking into account the control of conditions, renal biopsy is recommended.^140^

Considering the high specificity of serum PLA2R antibodies in patients with PMN, it is recommended that PLA2R antibody‐positive patients with nephrotic syndrome undergo dynamic testing first instead of immediate renal biopsy.^141^, ^142^ It is worth noting that if the patient has bleeding risk (platelets < 120 × 103/μL, increased INR or uses anticoagulant drugs), high blood pressure (systolic blood pressure >140 mmHg), or low renal function (eGFR <30 mL/min/1.73 m^2^), puncture site infection or persistent pyelonephritis and other relative contraindications to renal biopsy, these problems need to be intervened first.^124^

### 3.4 Diagnosis via novel target antigens

Due to the research on the mechanisms of MN over the past 20 years, a number of novel target antigens and autoantibodies associated with this disease have been identified (for example: PLA2R,^18^ THSD7A,^20^ EXT1/EXT2,^49^ etc.). The MN‐specificity of these antigens has made it possible to use serological testing as a new diagnostic method.^143^ In contrast to the traditional diagnostic method of renal biopsy, using commercial kits to detect antibody levels of specific antigens is less invasive and less expensive for the patient. Serum PLA2R antibodies have a positivity rate of more than 70% in patients with PMN,^31^ and supplemented by other target antigens found in the serum, it has now become a good tool to assist renal biopsy for diagnosis.^48^, ^49^, ^108^ Commercial kits for detecting serum PLA2R antibodies are already used in clinical practice.^144^

However, whether PLA2R can replace renal biopsy as preferred diagnostic standard remains questionable.^145^ Novel target antigens and autoantibodies identified in PMN have also appeared in SMN caused by malignant tumors or autoimmune disease.^146^, ^147^, ^148^ Meanwhile, due to the unique clinical and pathological phenotypes of many forms of MN associated with novel MN target antigens, Mayo Clinic suggests that a new classification of MN could be proposed based on novel target antigens.^60^, ^61^ Whether the new classification could improve the specificity and sensitivity of target antigen detection and guide treatment more effectively requires further investigation.

## 4 TREATMENT STRATEGIES FOR MN

Currently, the treatment approach for patients with MN is highly individualized, with experts recommending a conservative regimen tailored to each patient's specific complaints, clinical manifestations, and level of proteinuria. The primary goal of this regimen is to alleviate clinical symptoms such as proteinuria, edema, and hypertension. It is worth noting that approximately one‐third of patients experience spontaneous resolution of symptoms following supportive therapy. However, for patients who do not achieve remission or are at high risk of disease progression, immunosuppressive or combination therapy is commonly employed. In recent years, targeted therapy has emerged as a promising and more precise treatment modality. This approach not only complements or replaces traditional immunosuppressive therapies but also provides a novel avenue for personalized treatment. In the subsequent section, we will delve into a comprehensive discussion of the advantages and disadvantages of these therapeutic strategies, as well as their applicability in clinical practice.

### 4.1 Role of supportive care and lifestyle modifications in MN treatment

Dietary adjustments and regular physical activity play crucial roles in normalizing body mass index, minimizing central obesity, and mitigating cardiovascular disease risk factors. The occurrence of edema in MN hinges on sodium retention, with edema potentially leading to mobility issues, fatigue, malaise, infections, and anxiety.^149^ A low‐salt diet is recommended when edema is present, with patients advised to restrict sodium intake to 2.0 g/day. Depending on the level of proteinuria and renal function, adults may consider moderating dietary protein, typically limiting intake to 0.8–1 g/kg/day.^28^, ^150^ To address hyperlipidemia, reducing saturated fatty acids in favor of polyunsaturated fatty acids and incorporating soluble fiber into the diet is advised.

Irrespective of the degree of proteinuria and renal dysfunction in patients, the 2021 Kidney Disease: Improving Global Outcomes guidelines^151^ advocate for the routine conservative management of all individuals with MN, aiming to reduce morbidity and mortality.^150^, ^152^ When employing diuretic therapy in these patients, caution is advised to avoid overly rapid or aggressive regimens, as these approaches may lead to hypovolemia, exacerbate blood hyperviscosity, and precipitate thrombotic and embolic complications. Gradual dose escalation of diuretics is recommended, with a preference for loop diuretics over thiazides, until the maximum tolerable dose is reached or edema resolves. In cases of diuretic resistance, alternative diuretics with different mechanisms of action or even ultrafiltration may be considered.

The guidelines endorse angiotensin‐converting enzyme inhibitors (ACEIs) or angiotensin II receptor blockers (ARBs) as the primary pharmacological agents for managing hypertension and proteinuria in MN, with dosages titrated to the maximum effective or tolerable levels. Mild increases in serum creatinine (<30%) do not necessitate discontinuation of ACEI or ARB. Blood pressure targets for adults are set at< 120/80 mmHg, with the goal of reducing proteinuria to <1 g/day. Lowering proteinuria and boosting albumin levels may help prevent infections, thromboembolism, and renal function decline. Intravenous albumin administration is recommended when serum albumin falls below 2.0 g/dL.^153^

Statins are the preferred initial medication for managing dyslipidemia in MN patients, with alternative interventions considered for those who are intolerant to statins. Patients experiencing thromboembolic events in the setting of MN should receive full anticoagulation therapy. Prophylactic anticoagulation is warranted when the risk of thromboembolism in nephrotic syndrome patients outweighs the patient‐specific risk of significant bleeding events associated with anticoagulation.

### 4.2 Immunosuppressive therapies for MN

**FIGURE 5:**
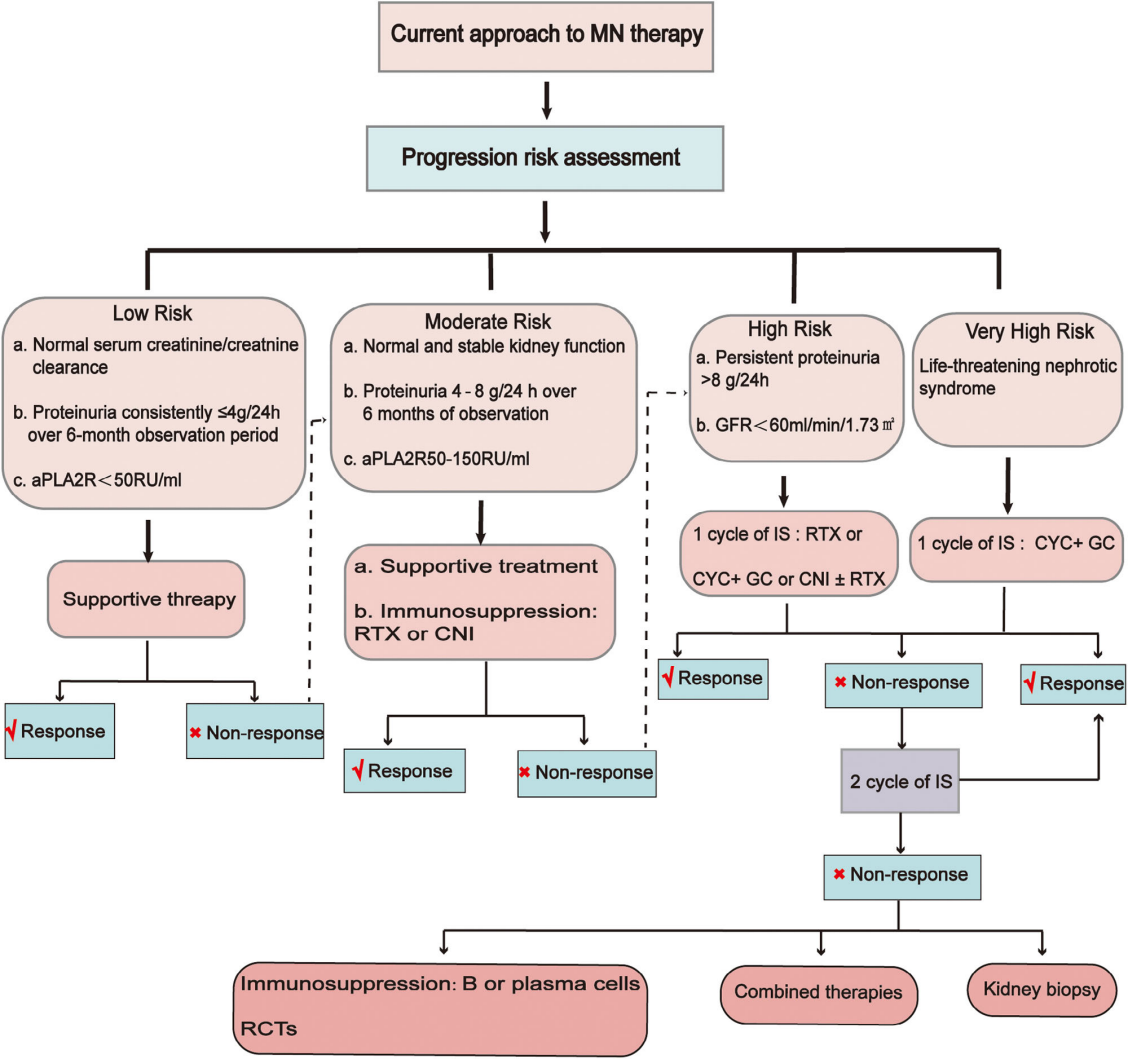
Grading and treatment of MN. CYC, cyclophosphamide; GC, glucocorticoids; RCTs, Randomized controlled trails.

The 2021 KDIGO guidelines delineate four risk categories for classifying MN: low, intermediate, high, and very high risk (Figure 5). While most patients’ disease characteristics may not precisely align with a single category and the risk classification may not be highly precise, it still offers valuable guidance for patient management. Risk assessment is an ongoing process, and it is crucial to re‐evaluate risk prediction at 3 and 6 months postdiagnosis, as alterations in PLA2R antibody levels and clinical parameters could impact therapeutic decisions.^154^

The treatment approach for MN must be tailored based on the patient's individual risk factors. Initially viewed as an immune‐mediated kidney disorder before the discovery of pathogenic antigens and specific autoantibodies, MN was commonly managed with prednisolone and other immunosuppressive medications. A randomized controlled trial demonstrated that a combination of an alkylating agent (preferably cyclophosphamide) and prednisolone could slow down the progression of renal failure in MN patients.^155^ Nevertheless, the 2012 KDIGO guideline cautions against the widespread use of such immunosuppressive therapy in MN patients. Due to the significant incidence of adverse effects associated with cyclophosphamide and corticosteroids, along with the observed spontaneous remission of proteinuria in about 40% of patients, the guidelines recommend restricting cyclophosphamide‐based therapy to MN patients at high risk of renal failure.^156^

The introduction of novel immunosuppressive agents holds the potential for providing effective and less toxic treatment options for MN patients. Calcium‐modulated phosphatase inhibitors like cyclosporine and tacrolimus indirectly modulate B cell function and have demonstrated efficacy in preventing immune rejection postrenal transplantation. Studies have revealed that these inhibitors can directly target podocytes, leading to a reduction in proteinuria.^157^ Moreover, the advent of CD20 antibody therapies, such as rituximab, has been proven effective in depleting B cells and selectively addressing the production of pathogenic antibodies. Additionally, CD20 antibody therapies, including rituximab, have shown effectiveness in preventing immune rejection following renal transplantation.^158^

The 2021 KDIGO guidelines recommend two treatment options for MN. For patients at intermediate risk, the recommendation includes treatment with rituximab or a calcineurin phosphatase inhibitor with or without glucocorticoids, closely monitoring the patient's condition. For those at high risk, the guidelines suggest treatment with rituximab or cyclophosphamide alternating with glucocorticoids, or a calcineurin phosphatase inhibitor with rituximab. Meanwhile, for patients at very high risk, the recommended treatment involves the use of cyclophosphamide and glucocorticoids.

### 4.3 Future directions in treatment of MN

Extensive research has indicated that patients affected by MN possess a dysregulated immune system,^159^, ^160^ characterized by aberrant expression of B‐lymphocyte subsets and B‐cell related factors.^23^ These findings strongly suggest the pivotal role played by B‐lymphocytes in the pathogenesis of MN. Notably, CD20,^161^, ^162^ a transmembrane phosphoprotein prominently present on the surface of B cells, represents a promising therapeutic target for the treatment of MN. In 2000, the treatment of kidney disease saw the emergence of Tuximab (RTX), a chimeric anti‐CD20 chimeric immunoglobulin.^163^ This therapeutic agent selectively targets B‐cells^28^, ^128^ and safeguards the functionality of glomerular podocytes.^164^ Furthermore, studies have showcased its capacity to reverse epitope spread.^165^, ^166^ After undergoing RTX treatment, a significant majority of individuals with IMN experience either complete or partial remission (CR or PR),^167^ with an impressive overall remission rate of 83.12%.^168^ Remarkably, it has demonstrated superiority over cyclosporine in sustaining the remission of proteinuria.^169^ In certain cases, repeated infusions of rituximab are necessary to more effectively deplete the PLA2R antibody.^170^ While rituximab is deemed safe and efficacious for patients with MN,^171^, ^172^ there are limitations to its effectiveness as 20–40% of patients do not respond.^173^ This lack of response may be attributed to higher PLA2R antibody titers, thus necessitating the exploration of alternative therapies with enhanced efficacy. Over the past decade, two new drugs, Obinutuzumab^174^, ^175^ and Belimumab,^176^, ^177^ have been discovered. These drugs target different epitopes on CD20 compared with RTX. Obinutuzumab induces B‐cell apoptosis^178^ and accelerates the depletion of PLA2R antibodies,^179^ resulting in >60% remission of proteinuria.^180^, ^181^ On the other hand, Belimumab reduces B‐cell survival.^182^ Both of these innovative monoclonal antibodies targeting CD20 hold promise as better treatment options for patients with high‐risk or very high‐risk MN.

In addition to CD20, CD38 has recently emerged as a potential therapeutic target for the management of MN. Daltuzumab and felzartamab, pioneering anti‐CD38 agents, hold promise in this regard, with daltuzumab exhibiting the ability to elicit programmed cell death via fc‐γ receptor‐mediated cross‐linking,^183^ though its efficacy necessitates further examination. Moreover, the intervention of belimumab,^184^ targeting B lymphoid stimulating factor (BlyS), brings about the apoptosis of autoreactive B cells and showcases favorable outcomes for individuals afflicted with systemic lupus erythematosus,^185^ thereby offering a novel treatment strategy for MN. Notably, Zhang et al.^186^ have recently discovered that telithromycin encompasses comprehensive suppression of CD20‐positive B cells, plasma cells, as well as T cells, thus instilling new‐found optimism for both MN and refractory MN management. Nonetheless, prolonged utilization of immunosuppressive drugs may give rise to substantial ramifications, encompassing organ toxicity, malignant tumors, and severe opportunistic infections.^187^ Consequently, redirecting our therapeutic approaches from indiscriminate immunosuppression towards antigen‐specific interventions stands to curtail reliance on immunosuppressive drugs, thereby mitigating their attendant side effects and signifying a momentous stride in the realm of MN treatment.

Potential targets of antigen‐specific therapeutic regimens encompass autoantibody production, antibody–antigen interactions, and immune‐mediated podocyte injury.^188^ It has been revealed that selective eradication of PLA2R antibodies through immunosorbent therapy diminishes disease activity.^189^, ^190^ While this constitutes a secure and widely embraced methodology for PLA2R‐positive patients, it falls short of offering a definitive cure for the ailment.^190^ Consequently, impediment of antibody production assumes pivotal significance. Employing T‐cell therapy incorporating chimeric antigen receptors (CARs)^191^, ^192^ stands as a prevalent strategy, enabling genetically engineered T cells to directly adhere to aberrant cell clusters, thereby annihilating such deranged entities by recognizing specific epitopes linked to the pathogenic antigen. Moreover, this approach holds potential for engendering memory CAR‐T cells, affording a sustained therapeutic effect.^193^ Alongside CAR‐T cell therapy, alternative cellular immunotherapies that harness antigenic epitopes, including T‐cell receptor gene therapy and checkpoint inhibitor therapy, have also demonstrated promise. Furthermore, advances have been made in investigating the prospects of devising vaccines and therapeutics incorporating diminutive peptide‐interfering antibodies that selectively bind to the PLA2R antigen. Last, the study conducted by Miao et al.^194^ has unveiled, for the first time, the ability of Sirt6 to forestall podocyte injury by impeding the RAS pathway via inhibition of the Wnt1/β‐catenin cascade. Sirt6 thus emerges as a potential therapeutic target for the treatment of nephropathy associated with podocyte injury.^194^

**FIGURE 6:**
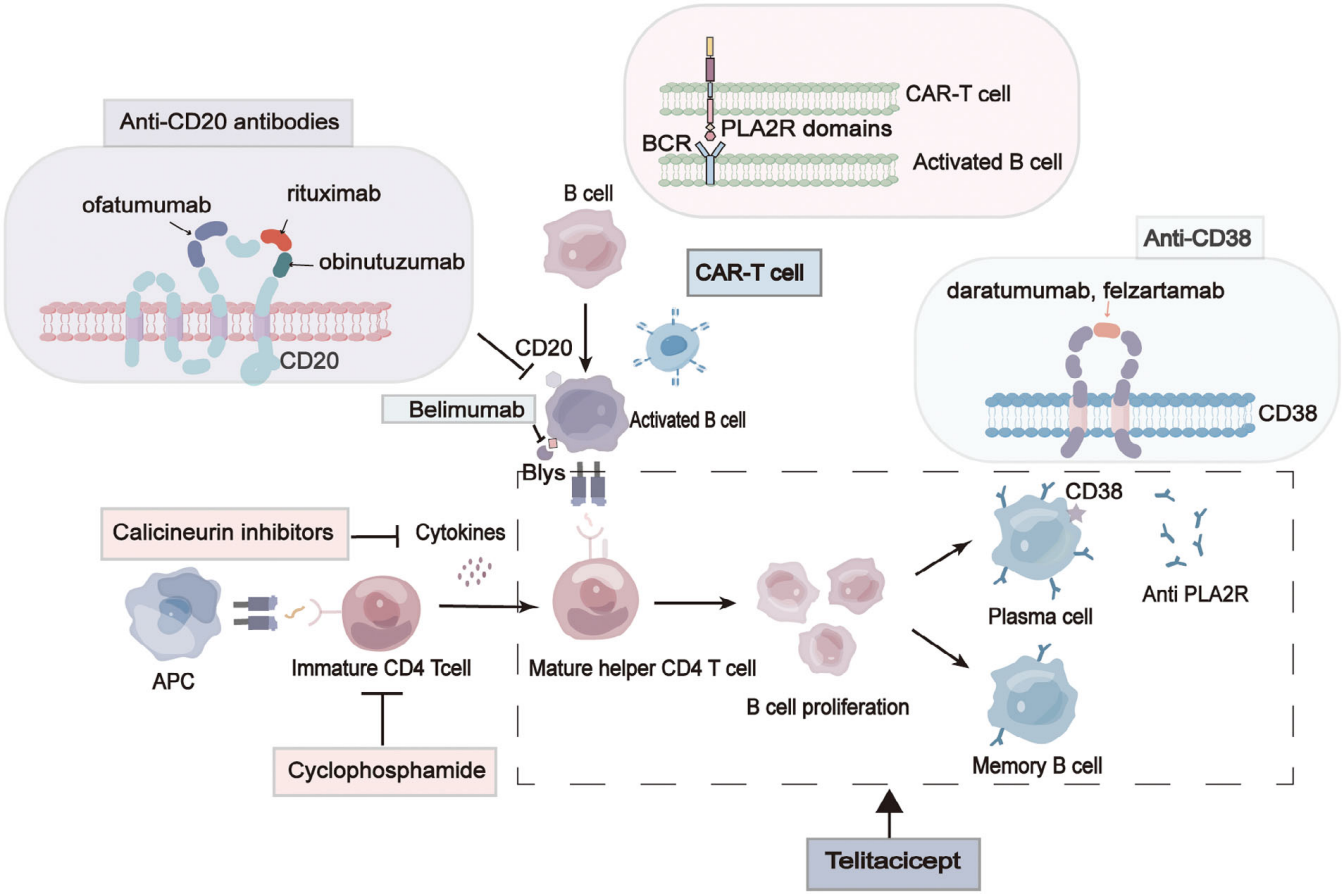
Current and future therapies for MN. Therapeutic approaches for membranous nephropathy encompass a repertoire of classical immunosuppressive therapies as well as innovative, targeted treatments. Affinity‐matured B cells undergo a metamorphosis, transitioning into either memory B cells characterized by the CD20 marker or CD20/CD38 plasma cells, which stand as key sources of antibodies and autoantibodies. The impact of cyclophosphamide primarily manifests in hampering the activation and proliferation of T cells and early B cells. On the other hand, calcineurin inhibitors (CNIs) not only impede the differentiation and proliferation of T cells but also elicit a reduction in glomerular PLA2R expression. Moreover, through hemodynamic mechanisms and the stabilization of podocyte structures, CNIs mitigate proteinuria. Anti‐CD20 agents assume responsibility for inducing the demise of CD20+ B cells at pivotal stages, thereby curbing the progression towards advanced B cells and plasma cells while concurrently diminishing autoantibody production. Belimumab, on the other hand, selectively targets B‐cell activating factor (BAFF), consequently leading to the apoptosis of B cells, arresting their transformation into plasma cells, and ultimately reducing autoantibody production. Notably, anti‐CD38 medications place their focus on plasma cells, effectively diminishing the production of autoantibodies, particularly in cases that have relapsed or proven refractory.

Bortezomib, a proteasome inhibitor approved by the United States Food and Drug Administration for the treatment of multiple myeloma, acts by inhibiting the transcription factor nuclear factor‐κB and depleting the ADAMTS13 antibody in thrombotic thrombocytopenic purpura.^195^ Its mechanism of action involves plasma cell depletion through the accumulation of misfolded proteins participating in cellular growth and differentiation. Notably, bortezomib exhibits mechanistic similarities to rituximab in the removal of autoantibodies, although it distinguishes itself by clearing pathogenic antibody‐producing cells or plasma cells, whereas rituximab solely targets B cells.^196^ Additionally, case reports have demonstrated that bortezomib usage in refractory MN has resulted in immune and clinical remission. Furthermore, bortezomib treatment has been linked to a reduction in the number of cells producing autoantibodies^197^, ^198^, ^199^ (Figure 6).

Iptacopan,^200^ a selective factor B inhibitor that targets the alternative complement pathway, is currently under evaluation in a randomized controlled trial comparing its efficacy with rituximab in patients with PLA2R‐positive MN (NCT04154787). On the other hand, narsoplimab,^201^ a humanized IgG4‐lambda monoclonal antibody serving as a recombinant Mannose Associated Serine Protease 2 inhibitor for the lectin pathway, is undergoing clinical trials to assess its effectiveness in various glomerular diseases, including MN (NCT02682407). A study investigating the effectiveness of plasma exchange in combination with rituximab therapy for MN patients unresponsive to standard treatment showed that 90% of patients achieved PR, with a mean urinary protein/creatinine ratio of 1.1 g/g.^202^ Furthermore, a multicenter, single‐arm clinical trial examined the efficacy of peptide GAM immunoadsorption in selectively removing IgG subclasses in 12 patients with MN. Despite a decrease in PLA2R titers postimmunoadsorption, the clinical impact was not significant, and there was a subsequent rise in PLA2R titers during follow‐up.^190^ Consequently, the effectiveness of these in vitro therapeutic approaches remains uncertain, emphasizing the necessity for large‐scale studies to evaluate the outcomes of these adjunctive strategies.

With in‐depth research on the immunology, pathophysiology, pathology, symptoms and clinical manifestations, and biomarkers of MN, future treatment options for MN will be more precise and diversified. The development of novel immunosuppressants and immunomodulators provide more possibilities for drug selection, aiming to reduce side effects and improve patients' quality of life and long‐term prognosis. More potential target proteins associated with MN have been identified, and antigen‐specific therapeutic regimens against PLA2R antibodies, the major autoantibodies in MN, are likely to be further optimized, setting the stage for rapid development and mature individualization of targeted therapies in the future. Additional clinical studies will explore the combinations of different drugs, such as treatments that combine existing immunosuppressants with new drugs or targeted therapies, to improve efficacy and minimize side effects. There are still a number of pathways in basic research, including inhibition of inflammatory factors, modulation of cell adhesion molecules, and intervention in the complement system, and so on, which are expected to be transformed into new treatments. Regenerative medicine and stem cell technology may also be explored in the future for repairing damaged GBMs or restoring kidney function. Even the currently hot cross‐species organ transplantation techniques, such as pig kidney transplantation, has shown unprecedented possibilities and hopes for patients with end‐stage MN.

## 5 COMPLICATIONS OF MN

### 5.1 Thrombotic complications and treatment

Various complications of NS are often also reflected in the development of MN, such as thrombosis and embolism, hypertension, hyperlipidemia, and so on, which greatly affect the patients’ treatment strategy and prognosis, and reduce their quality of life.

In a retrospective cohort study of patients with NS, the annual incidence of venous thromboembolism (VTE) or arterial thromboembolism (ATE) was found to be higher in the NS population than in the general population (1 and 1.5% annual incidence of VTE and ATE, respectively, in the NS population), and the risk was significantly elevated in the first 6 months after diagnosis (9.85 and 5.52%, respectively).^203^ Among nephrotic syndrome‐related diseases with a significantly increased risk of thrombosis, MN has a higher incidence of thrombosis and embolism than other kidney diseases, among which renal vein thrombosis, lower extremity vein thrombosis, and pulmonary embolism are the most common,^204^, ^205^, ^206^ and ATE or other possible venous involvement also occurs.^203^, ^207^

The hypercoagulable state of the blood in patients with MN may contribute to the susceptibility to thrombotic events. An imbalance in coagulation homeostasis caused by decreased levels of antithrombotic factors (loss of antithrombin III and plasminogen)^208^ versus increased levels of procoagulant factors (increased synthesis of factor VIII, increased fibrinogen)^209^ may be the main cause of this states. Abnormal platelet activation and aggregation,^210^ hypoalbuminemia,^211^ side effects of hormone therapy, decreased blood volume by diuretic use, and gender^212^, ^213^ are also important influences contributing to the blood hypercoagulability. Since MN is an immune nephropathy, relevant research on the impact of immune^214^, ^215^, ^216^ and environmental factors^217^ on the risk of thrombotic events is also ongoing.

The management of thrombotic complications in MN is mainly divided into two aspects: treatment and prevention. For patients who have developed thromboembolism, the recommended treatment is sequential anticoagulation with high or low‐molecular‐weight heparin followed by oral warfarin.^150^, ^204^ For patients who have not yet developed embolism, serum albumin levels are often used clinically as an independent risk predictor for a thrombotic event.^204^ When the serum albumin value is <20–25 g/L, it indicates a high risk of thrombotic events^218^, ^219^ and patients are advised to take follow‐up measures High‐risk patients require further bleeding assessment.

Referring to the KDIGO 2021 guidelines for the management of glomerular disease, some people with low bleeding risk can use high‐ or low‐molecular‐weight heparin and oral warfarin for anticoagulation, while other patients with high bleeding risk are recommended to use aspirin treatment.^140^, ^150^, ^220^ Aspirin prophylaxis may also be considered when the serum protein value of MN patients is <30 g/L and they also have other risk factors for thrombosis (such as congestive heart failure, prolonged sitting, morbid obesity, abdominal, orthopedic or gynecological surgery, family history of thrombotic tendency, etc.).^140^, ^150^ Although oral anticoagulants have the advantage of low bleeding risk, their pharmacokinetics are easily affected by decreased renal function and proteinuria. Whether they can become recommended antithrombotic drugs for MN is still under study.^221^, ^222^, ^223^

### 5.2 Other complications and treatment

In addition to thrombotic complications, patients with MN may have comorbid hypertension. Although hypertension is generally not severe in adult patients, it is still recommended that these people take ACEIs and/or ARBs agents to control blood pressure at a level of 125−130/75−80 mmHg.^118^, ^140^, ^224^ MN patients are often accompanied by hyperlipidemia, and abnormal lipid metabolism increases the risk of cardiovascular‐related diseases, including myocardial infarction, coronary heart disease, and thromboembolism.^225^, ^226^ Correlation studies with serum anti‐PLA2R antibodies, glomerular PLA2R deposition, and proteinuria findings in patients with PMN, as well as cases, suggest that hypercholesterolemia may be a potential biomarker for predicting the severity of PMN.^227^, ^228^ The guidelines therefore recommend that such patients routinely take statins for lipid lowering. Studies have shown that the use of statins can effectively reduce the occurrence of venous thromboembolic events^229^ (Table 1).

**TABLE 1:** Complications associated with membranous nephropathy.

Complication	Clinical presentation/classification	Treatment	Precaution	References
Infection (most common)	Common sites of infection: respiratory tract, urinary tract, digestive tract and skin	Anti‐infective drug without renal toxicity	Strengthen daily protection, improve immunity	1
Thrombosis and embolism	Deep vein thrombosis, lower limb vein thrombosis and pulmonary embolism were the most common	1. Anticoagulation (low‐molecular‐weight heparin, warfarin) 2. Antiplatelet (aspirin, clopidogrel) 3. Thrombolytic (urokinase, streptokinase)	Early preventive anticoagulation	151
Hypertension	Systolic blood pressure ≥130 mmHg, diastolic blood pressure ≥80 mmHg	Angiotensin converting enzyme inhibitors/angiotensin receptor blockers	Prevention of hyponatremia, daily monitoring of blood pressure	151
Lipid metabolism disorder	Total cholesterol and LDL cholesterol increased, and HDL cholesterol decreased	Cholesterol‐lowering, triglyceride lowering: Statins (Lovastatin)	Patients with persistent proteinuria and hypercholesterolemia, especially those >50 years of age, are treated with statins.	151
Acute kidney injury	Sudden decrease in glomerular filtration rate or new hematuria	1. Control blood sugar <10 mmol/L 2. The controlled protein intake for AKI patients who do not require dialysis is 0.8–1.0 g/kg d 3. Avoid nephrotoxic drugs	Renal function indicators (serum creatinine and urine volume) were continuously monitored and novel AKI markers, such as NGAL, were selected for auxiliary monitoring	151

### 5.3 End‐stage renal disease and replacement therapy

About 30% of patients with untreated MN can resolve spontaneously, and these patients have a good prognosis and a low recurrence rate.^26^, ^224^ However, some patients maintain nephrotic syndrome for a long time, and large amounts of proteinuria will lead to continued decline in renal function and eventually progression to end‐stage renal disease (ESKD).^230^ Currently, renal transplantation is the best treatment for ESKD, and usually results in the best patient outcomes.^231^However, renal transplantation is faced with a shortage of renal sources and high costs, and common posttransplantation problems (such as immunosuppression, rejection, disease recurrence, and infections, etc.), require a challenging and high level of medical management.^232^ Therefore, the majority of patients with ESRD choose to receive dialysis, and some of these populations experience a recovery of renal function after long‐term (approximately 10 months) dialysis.^231^, ^233^

Choosing the appropriate timing of dialysis requires not only consideration of the patient's GFR, but also customization to the individual needs of the patient in terms of age, gender, comorbidities (diabetes, hypertension, etc.), and physical condition.^234^, ^235^, ^236^

## 6 PROGNOSIS OF MN

The process of MN is slow so its prognosis is affected by many factors, including the severity of the disease itself, its comorbidities, response to treatment, age, gender, and so on. Therefore, the knowledge of reasonable methods for monitoring disease progression and assessing response to treatment will help to adjust treatment strategies in a timely manner and to achieve a better expected outcome.^237^

Currently, clinically validated criteria include proteinuria level, GFR,^238^ serum creatinine level,^239^ and urinary low‐molecular‐weight proteins such as uα2m and uβ2m.^240^ According to research, urinary protein level, that is, the degree of urinary protein loss, is closely related to prognosis. A >50% decrease in proteinuria from baseline in the first year after diagnosis is an important independent predictor of spontaneous remission.^224^ As an assessment indicator of overall renal function, a sharp decline in GFR leading to acute renal failure or a slow decline to end‐stage levels is a sign of significant poor prognosis.^241^ In addition, factors such as serum albumin and serum PLA2R antibody levels also have auxiliary significance in clinical application.^118^, ^242^, ^243^ The KDIGO 2021 guidelines use four risk levels to classify patients into four risk levels based on prognostic markers: low risk, intermediate risk, high risk, and very high risk, but there are no clear boundaries for each category.^140^

Improvement in the levels of these prognostic markers and patients who do not demonstrate significant adverse drug reactions are considered to have achieved therapeutic efficacy.^244^ In the clinical setting, good MN outcomes are reflected in CR and PR, with CR being defined as proteinuria ≤0.3 g/d and a stable GFR and PR being defined as a reduction in proteinuria of >50% and proteinuria <3.5 g/d and a stable GFR. The recurrence rate is higher in patients with PR than in those with CR.^230^ Patients with CR may relapse to subnephrotic levels (<3.5 g/d) with or without other symptoms of nephrotic syndrome, but rarely relapse completely to nephrotic levels (≥3.5 g/d); for patients with PR, relapse is usually defined as an increase in proteinuria to ≥3.5 g/d.^245^

## 7 CONCLUSIONS

MN, as the most common cause of NS in adults, has a persistent negative impact on patients' renal function and physical health without early medical intervention. According to the need for healthy aging in society, with the average age at diagnosis of MN is 50−60 years old, the impact of the disease itself and maintenance treatment on the patients’ quality of life also deserve attention. Therefore, in the face of clinical requirements for early diagnosis, improvement of refined and noninvasive diagnosis, diversified and individualized treatment, and maintenance of therapeutic effects, cutting‐edge research continues to deepen from the superficial symptoms and histology to the level of pathology, molecular biology and biochemistry. In the field, important breakthroughs have been made in terms of disease mechanisms and treatment targets.

In recent decades, significant discoveries have advanced our understanding of MN pathogenesis and its optimal clinical management. The identification of novel MN target antigens and autoantibodies has prompted the proposal of a new classification that integrates these elements into the pathogenesis of MN. The potential pathogenesis of HLA and novel pathogenic variants together with their interaction between target antigens are new directions for future research, which will help deepen our understanding of disease heterogeneity and provide new perspectives for the development of treatment options.

According to the 2021 KDIGO Guidelines, the standard of care for diagnosed MN emphasizes a multidisciplinary approach encompassing pharmacologic interventions, supportive measures, and antibody monitoring. Ongoing efforts to address refractory MN include multiple antigen‐specific treatment regimens and large‐scale clinical trials, the outcomes of which are expected to enhance evidence‐based treatment strategies for MN. Nonetheless, further research is necessary to assess disease diagnosis, monitoring, and treatment strategies, with the potential to advance the development of personalized therapies for MN.

At present, scientific research results that have reliable experimental and clinical basis need to be further evaluated and applied. For example, the antibody detection of PLA2R and THSD7A has become an important biomarker for the diagnosis of PMN. Next, the immunoassay method of markers needs to be further optimized and combined with other noninvasive diagnostic techniques to find a method with better specificity and sensitivity that can replace traditional renal biopsy diagnosis. At the same time, proven and reliable biomarkers, genetic factors, clinical symptoms, and biochemical indicators can be comprehensively considered to develop disease monitoring models or prognosis prediction models, providing a basis for the selection of subsequent treatment options and maintenance treatment. In addition to early diagnosis and identification of high‐risk groups, the study of pathological mechanisms is also a potential therapeutic target, which is expected to reduce the adverse reactions caused by traditional immunosuppressive treatment and improve the quality of life. Therefore, the new progress made in the research field in recent years needs to be more closely integrated with clinical practice in the future and transformed into excellent products that can actually meet the needs of clinicians and patients.

## AUTHOR CONTRIBUTIONS

Yi Yang and Weiqiang Lin have provided important guidance for this paper. Mengqiong Wang drafted the manuscript and completed the illustrations and descriptions. Jingjuan Yang and Xin Fang provided the main writing ideas and further refined the article. All authors have read and approved the final manuscript.

## CONFLICT OF INTEREST STATEMENT

All authors declare they have no conflict of interest.

## ETHICS STATEMENT

Not applicable.

## Data Availability

Not applicable.
